# Association mapping of susceptibility loci for rheumatoid arthritis

**DOI:** 10.1186/1753-6561-1-s1-s15

**Published:** 2007-12-18

**Authors:** Tai-Yue Kuo, Winston Lau, Cheng Hu, Weihua Zhang

**Affiliations:** 1 Human Genetics Division, Duthie Building (Mailpoint 808), Southampton General Hospital, Tremona Road, Southampton SO16 6YD, UK; 2 National Cheng Kung University Hospital, No. 138, Shengli Road, Tainan City, Taiwan; 3 Shanghai Diabetes Institute, Shanghai Jiaotong University, 600 Yishan Road, Shanghai 200233, People's Republic of China; 4 Section of Cancer Genetics, The Institute of Cancer Research, 15 Cotswold Road, Belmont, Sutton Surrey SM2 5NG, UK; 5 Department of Cardiology, Ealing Hospital NHS Trust, Uxbridge Road, Southall, Middlesex, UB1 3HW, UK

## Abstract

We analyzed a case-control data set for chromosome 18q from the Genetic Analysis Workshop 15 to detect susceptibility loci for rheumatoid arthritis (RA). A total number of 460 cases and 460 unaffected controls were genotyped on 2300 single-nucleotide polymorphisms (SNPs) by the North American Rheumatoid Arthritis Consortium. Using a multimarker approach for association mapping under the framework of the Malecot model and composite likelihood, we identified a region showing significant association with RA (*p *< 0.002) and the predicted disease locus was at a genomic location of 53,306 kb with a 95% confidence interval (CI) of 53,295–53,331 kb. A common haplotype in this region was protective against RA (*p *= 0.002). In another region showing nominal significant association (51,585 kb, 95% CI: 51,541–51,628 kb, *p *= 0.037), a haplotype was also protective (*p *= 0.002). We further demonstrated that reducing SNP density decreased power and accuracy of association mapping. SNP selection based on equal linkage disequilibrium (LD) distance generally produced higher accuracy than that based on equal kilobase distance or tagging.

## Background

Rheumatoid arthritis (RA) is a common chronic disease, with a moderately strong genetic component. Chromosome 18q has shown evidence for linkage in the U.S. and French linkage scans [1]. The North American Rheumatoid Arthritis Consortium (NARAC) performed fine mapping on a 10-Mb region on 18q with a dense single-nucleotide polymorphism (SNP) map and the data were collected by the Genetic Association Workshop (GAW) 15 for Problem 2. Here we applied a novel association mapping approach based on the Malecot model and composite likelihood to identify disease associated regions and predict the locations of possible disease loci [2]. Haplotype analysis on the candidate regions was performed. We also studied the effect of region length and SNP density on the accuracy of association mapping in comparison with our analysis of the simulated data in Problem 3 of GAW15 [3].

## Methods

### Data

A total of 2300 SNPs in a 9,519.224 kb region of 18q were genotyped by NARAC in 460 cases of RA and 460 controls. Controls were recruited from a New York City population. Seven SNPs showing significant departure from Hardy-Weinberg equilibrium (HWE) in the control samples using a likelihood ratio chi-square test (χ^2 ^≥ 10) were removed, resulting in a total of 2293 SNPs [4]. Further removal of 81 SNPs with a minor allele frequency (MAF) of less than 5% resulted in a total of 2212 SNPs for our main data analysis.

### LD map

Physical locations of these SNPs were determined from build 35 (UCSC May 2004) of the human genome sequence. An LD map expressed in linkage disequilibrium (LD) units (LDUs) was created using the control samples with the LDMAP-cluster program, a parallel version of LDMAP program that rapidly constructed the map [5]. LDU is determined by the product of the ε and distances in kilobases for an interval of two adjacent SNPs and is additive, where ε represents the exponential decline of LD with distance for that interval. The LD map length was 151.115 LDUs, which is essentially the same as the 2293 SNPs containing rare SNPs.

We also used the LD map built from the CEU samples of the HapMap Phase II data [5]. The same region on the CEU LD map contains 8086 SNPs with a length of 202 LDUs. Despite its higher SNP density, 185 SNPs were missing and therefore their LDU locations were linearly interpolated. However, alternative LD maps did not seem to exert a significant effect on the results (data not shown), and we hereby only report the results using the LD map constructed from the GAW15 data.

### Association mapping

The 10-Mb segment of 18q was divided into 14 non-overlapping consecutive regions. Each region had a minimum of 10 LDUs and 30 SNPs by default without breaking LD blocks and was analyzed individually. We also used a 5-LDU region length, resulting in a total of 26 regions for association analysis. In the Malecot model, association is a function of several parameters, the most important of which is *S*, the predicted location of the disease variant [2]. Composite likelihood combines information of all pairwise marker-disease associations in each region. *S *and its 95% confidence interval (CI) are estimated by fitting the model to the data and maximizing the composite likelihood. Significance tests are carried out by contrasting two hierarchical models. Model A assumes no association and no parameters are estimated. Model D assumes an association and *S *and two other parameters are estimated with ε specified. The difference in the -2 natural log composite likelihood (denoted as Λ) between the two models (denoted as Λ_A _- Λ_D_) is a statistic monotonic to a chi-square with 3 degrees of freedom ($\chi_{3}^{2}$). A permutation test was performed for each region with hundreds of replicates under the null hypothesis of no association by shuffling case-control status to obtain an empirical *p*-value [2]. The specified value for ε of 1.0543 was obtained by fitting the LD map to the genotype data for the control samples. However, similar values for ε did not seem to have an appreciable effect on the results (data not shown).

This approach has been implemented in the CHROMSCAN program and a parallel version, CHROMSCAN-cluster, based on cluster computing was used for permutations with 1000 replicates . Pearson's χ^2^s were obtained for allelic associations between single SNPs and RA.

### Haplotype analysis for candidate regions

Haplotype analysis using the PHASE program version 2 [6] was performed for candidate regions showing nominal significant association. The five most common haplotypes and their frequencies were compared between cases and controls. A chi-square test was applied to identify significant associations by testing each haplotype in turn against all others, including rare ones.

### SNP density and region length

To generate different SNP density, we used Tagger in Haploview software to select tagging SNPs based on pairwise LD (*r*^2^) using the control samples with rare SNPs included [7]. For comparison, we selected the same number of SNPs as Tagger but by equidistance in LDU or kilobases. To do this, SNPs with the same LDU were reassigned LDU locations by linear interpolation (tilting) so that every SNP would have a unique location. By centering at the "disease locus", we also studied the effect of LDU region length on the results.

## Results and discussion

### Association mapping of disease locus

**Figure 1:**
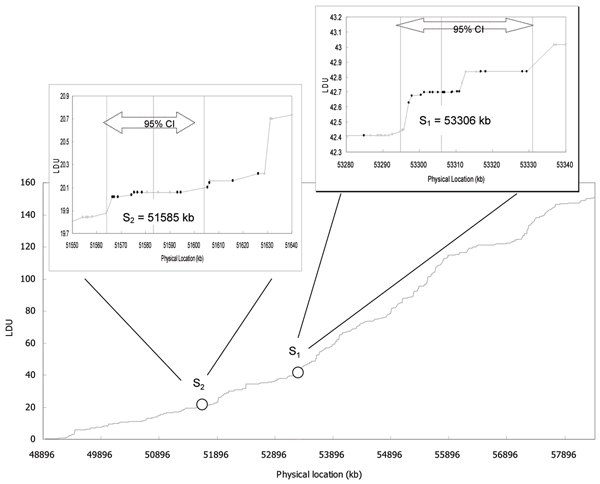
**An LD map in relation to the putative disease loci *S*_1_and *S*_2_**. The details of the SNPs and LD patterns for the regions around *S*_1 _and *S*_2 _are enlarged in the upper diagrams. The vertical black solid line indicates the location of the point estimate within the 95% CI. The black dots on the map represent SNPs showing nominal significant association with RA (*p *< 0.05) and the gray dots represent SNPs showing no association.

We found a nominally significant association between region 5 and RA. The estimated location of the disease locus *S*_1 _was at 53,306 kb near a SNP of global maximal chi-square (rs3745064, χ^2 ^= 12.25, *p *= 0.00047) using the LD map with a 10-LDU region length (*p *= 0.002). After Bonferroni correction for 14 regions the results were still statistically significant (*p*_c _= 0.02). Removing this SNP did not change the results. However, inclusion of SNPs with MAF < 5% resulted in a wider 95% CI (53,274–53,342 kb, point location at 53,307 kb). Figure 1 shows that the estimated location for the disease variant was in a 10-kb LD block where a cluster of SNPs showed modest association with RA. At such a significance level, none of the SNPs were statistically significant after correction for multiple comparisons. This is in contrast to multimarker approaches, in which one cluster of SNPs is considered at a time in light of LD among nearby SNPs, markedly reducing the number of tests.

At 5-LDU region length with a total of 26 regions, we found a locus (*S*_2_) at 51,585 kb showing nominal significant association (*p *= 0.04, Table 1 and Figure 1). Similar results were obtained using the data with rare SNPs (MAF < 5%) included. In this region there was also a cluster of SNPs associated with RA. However, consideration of multiple testing this region would not be statistically significant.

**Table 1:** Association mapping of RA susceptibility loci

Loci	LDU length	No. of regions	Region (no. of SNP)	Length (kb)	Location *S *(kb)	95% CI (kb)	$\chi_{3}^{2}$	*p*
*S*_1_	10.2	14	5^th^(112)	371	53,306	53,295–53,331	15	0.0017
*S*_2_	5.1	26	4^th^(132)	703	51,585	51,541–51,628	8	0.0370

### Haplotype analysis for candidate regions

Haplotype analysis was performed on sub-regions containing *S*_1 _and *S*_2 _with the majority of nominally associated SNPs included. *S*_1 _sub-region (53,297–53,312 kb, 0.043 LDUs) contained 16 SNPs (Table 2) and *S*_2 _sub-region (51,556–51,616 kb, 0.368 LDUs) contained 21 SNPs (Table 3). Haplotypes *H*_1 _and *H*_3 _at *S*_1 _and *H*_5 _at *S*_2 _appeared to be significantly associated with RA, with the first two haplotypes being almost complementary. Both *H*_1 _at *S*_1 _and *H*_5 _at *S*_2 _showed protective effects against RA.

**Table 2:** Common haplotype analysis of the *S*_1 _candidate region

Code	*S*_1 _Haplotype^a^	Frequency			χ^2^	*p*
				
	+ + + + + + - + + + + + + + + -	Total	Case	Control		
*H*_1_	2 1 1 2 1 2 1 1 1 2 1 1 2 1 1 1	0.661	0.628	0.695	9.22	0.002
*H*_2_	1 2 2 1 2 1 1 2 2 1 2 2 1 1 2 1	0.169	0.180	0.158	1.59	0.2
*H*_3_	1 2 2 1 2 1 1 2 2 1 2 2 1 2 2 1	0.101	0.120	0.082	7.32	0.007
*H*_4_	1 1 1 2 1 2 1 1 1 2 1 1 2 1 1 1	0.019	0.013	0.025	3.55	0.06
*H*_5_	1 1 2 1 2 2 2 2 2 1 2 2 1 2 2 2	0.017	0.017	0.017	0.00	1

^a^SNPs from left to right: rs660936, rs674849, rs615030, rs629737, rs519596, rs660626, rs3745070, rs3745064, rs3848516, rs608017, rs608823, rs552396, rs2279096, rs1217583, rs3899444, rs4940796, '+', '-' denote a SNP with nominal association (+) or no association (-) with RA. '1', '2' denote the alleles of a SNP.

**Table 3:** Common haplotype analysis of the *S*_2 _candidate region

Code	*S*_2_Haplotype^a^	Frequency			χ^2^	*p*
				
	- - - - + + + + - + + + - - - + + + + - +	Total	Case	Control		
*H*_1_	1 2 1 2 2 1 1 1 1 2 2 1 2 2 1 2 2 1 2 2 2	0.339	0.343	0.336	0.10	0.8
*H*_2_	2 1 2 1 2 1 1 1 1 2 2 1 2 2 2 1 2 2 1 1 2	0.256	0.274	0.238	3.13	0.08
*H*_3_	2 1 2 1 2 1 1 1 1 2 2 1 2 2 1 2 2 1 2 1 1	0.100	0.089	0.110	2.26	0.1
*H*_4_	2 1 2 1 1 2 2 2 1 1 1 2 2 2 1 2 1 1 2 2 1	0.079	0.074	0.084	0.63	0.4
*H*_5_	1 2 1 2 1 2 2 2 1 1 1 2 2 2 1 2 1 1 2 2 1	0.075	0.056	0.094	9.57	0.002

^a^SNPs from left to right: rs813043, rs784254, rs711745, rs784251, rs4800995, rs784237, rs796743, rs784235, rs784233, rs4800996, rs3745044, rs784232, rs1642295, rs784240, rs1362781, rs2306163, rs931040, rs4996482, rs899101, rs899102, rs1031830, '+', '-' denote a SNP with nominal association (+) or no association (-) with RA. '1', '2' denote the alleles of a SNP.

Further analyses of *S*_1 _categorized all individuals into three groups, *H*_1_/*H*_1_, *H*_1_/*H*- and *H*-/*H*-, where *H*- is a haplotype other than *H*_1_. There was a significant association between haplotype pairs and disease status ($\chi_{2}^{2}$ = 10.3, *p *= 0.006). An individual carrying *H*_1_/*H*_1 _had a lower risk of RA than those carrying *H*_1_/*H*- or *H*-/H-. The odds ratios (ORs) were 0.58 (95% CI: 0.37–0.92) and 0.85 (0.54–1.34) for an individual carrying *H*_1_/*H*_1 _or *H*_1_/*H*- compared to *H*-/*H*- haplotypes, respectively. We performed analyses conditional on whether an individual carried *H*_1_/*H*_1_. Interestingly, *H*_5 _appeared to be significant only in *H*_1_/*H*- or *H*-/*H*- carriers (χ^2 ^= 7.647, *p *< 0.05), but not in *H*_1_/*H*_1 _carriers (χ^2 ^= 1.509), indicating a possible interaction between the two haplotypes.

### Genes and mRNA at the candidate regions

The UCSC genome browser (May 2004) was used to find genes and mRNAs within the 95% CI of loci *S*_1 _and *S*_2_. No known genes have been found nearby *S*_1_, but the area of the 95% CI for locus *S*_1 _contains four human mRNA (CR590917, AK021717, AK124558, and BC013134), two of which span the point estimate of *S*_1_. Therefore, this region might contain genes not yet identified. In addition, this area is highly conserved across species, implying functional importance of the genomic sequence. A known gene (*AK127787*) is within the 95% CI of *S*_2_, but is 10 kb away from its point estimate.

### Region length and SNP density

Point estimates of *S *were identical for all region lengths centred at *S*_1_. The 95% CI was also relatively stable, with a slow increase with region length (Table 4). Enlarged region length compromised the significance levels, perhaps due to noise from distant SNPs, given that the informative SNPs were clustered in a rather small region. Computing time prolonged with increasing number of SNPs. Small region lengths, however, resulted in a heavy penalty for multiple testing. Four LDUs provided the most significant result for *S*_1_(*P*_*c *_= 30 × 0.0008 = 0.02, Table 4).

**Table 4:** The impact of region length on association mapping

Region length		No. of regions	No. of SNPs in the region		$\chi_{3}^{2}$	*p*	Length of 95% CI (kb)
					
LDU^a^	kb		All	*p *< 0.05 (%)			
1	75	58	49	22 (45)	14.02	0.0029	34
4	122	30	66	27 (41)	16.83	0.0008	40
10	578	14	191	34 (18)	10.44	0.0152	73
20	1262	7	382	37 (10)	6.75	0.0802	76
60	3732	2	946	84 (9)	7.58	0.0554	76

^a^LDUs centering *S*_1 _at 53,306 kb to which all *S *estimates were equal.

Our analysis of simulated data indicated that reduced SNP density decreased mapping accuracy, and SNP selection based on equal LD distance produced smaller location errors than that based on equal kilobase distance or tagging [3]. Interestingly, among the three selection approaches for *S*_1 _region, Tagger selected the most number of SNPs while equal kilobase distance, the least number. SNPs selected by equal LDU distance generally provided the highest location accuracy (Table 5). Power was reduced with decreasing density, as indicated by the values of Λ_A _- Λ_D _(Table 5). Using the kilobase map resulted in higher location errors in most cases and lower Λ_A _- Λ_D _values, indicating reduced power in all circumstances compared with using the LD map (data not shown).

**Table 5:** SNP density and accuracy – selection by tagging or equidistance

*r*^2^/LDU/kb (kb/SNP)	No. of SNPs^a^	Location error^b^			Λ_A _- Λ_D_		
			
		Tagger	E_LD	E_kb	Tagger	E_LD	E_kb
Full (4)	189/189/189	0	0	0	79	79	79
1.0/0.002/1 (5)	160/163/150	1	0	-2	62	69	68
0.8/0.036/5 (11)	78/74/64	-14	4	-5	21	18	20
0.6/0.071/9 (15)	59/55/48	-26	-37	-3	10	11	10
0.4/0.134/14 (21)	43/39/33	-27	25	-5	12	11	8
0.2/0.300/26 (33)	28/21/20	-35	33	-180	7	9	1

^a^For the studied region for Tagger, Equal LD (E_LD) and kb (E_kb) distance, respectively.

^b^Assuming *S*_1 _is the "disease locus," the region was fixed at 10 LDUs centering at *S*_1 _and the location error was calculated as *S*-53,306 kb.

## Conclusion

We reported a significant association between a region of 18q and RA. The estimated genomic location of the disease variant was at 53,306 kb. The Malecot model and composite likelihood approach has narrowed the possible disease locus to a 36-kb candidate region. A haplotype significantly associated with reduced risk of RA was identified in this region. DNA sequences between 53,295–53,331 kb of this region are highly conserved in vertebrates. A haplotype around 51,585 kb was also identified as reducing the risk of RA. Further sequencing or functional studies may be helpful to identify the disease variants. Reducing SNP density decreases power and location accuracy. We also conclude that SNP selection based on equal LD distance can maximally retain the prediction accuracy of the disease loci than that based on equal physical distance or SNP tagging.

## Competing interests

The author(s) declare that they have no competing interests.

## References

[B1] Choi SJ, Rho YH, Ji JD, Song GG, Lee YH (2006). Genome scan meta-analysis of rheumatoid arthritis. Rheumatology (Oxford).

[B2] Morton NE, Maniatis N, Zhang W, Ennis S, Collins A (2007). Genome scanning by composite likelihood. Am J Hum Genet.

[B3] Zhang W, Lau W, Hu C, Kuo T-Y Impact of marker density on the accuracy of association mapping. BMC Proc.

[B4] Gomes I, Collins A, Lonjou C, Thomas NS, Wilkinson J, Watson M, Morton N (1999). Hardy-Weinberg quality control. Ann Hum Genet.

[B5] Lau W, Kuo TY, Tapper W, Cox S, Collins A (2007). Exploiting large scale computing to construct high resolution linkage disequilibrium maps of the human genome. Bioinformatics.

[B6] Stephens M, Smith NJ, Donnelly P (2001). A new statistical method for haplotype reconstruction from population data. Am J Hum Genet.

[B7] Carlson CS, Eberle MA, Rieder MJ, Yi Q, Kruglyak L, Nickerson DA (2004). Selecting a maximally informative set of single-nucleotide polymorphisms for association analyses using linkage disequilibrium. Am J Hum Genet.

